# Processability Map of a Recyclable Thermoplastic for Structural Applications. Kinetics of Induction Period

**DOI:** 10.1021/acsomega.5c10999

**Published:** 2025-12-12

**Authors:** Sihem Zaidi, Daniel Sánchez-Rodríguez, Jordi Farjas, Raquel Verdejo, Miguel Ángel López-Manchado, Daniel Trias, Josep Costa

**Affiliations:** † GRMT, Materials Research Group and Thermodynamics, Polytechnic School, 16738 University of Girona, Campus Montilivi, Edif. PII, E17003 Girona, Catalonia, Spain; ‡ AMADE, Analysis and Advanced Materials for Structural Design, Polytechnic School, University of Girona, Campus Montilivi, Edif. PII, E17003 Girona, Catalonia, Spain; § Institute of Polymer Science and Technology (ICTP), CSIC, C/Juan de la Cierva 3, 28006 Madrid, Spain

## Abstract

In this study, we conducted a comprehensive kinetic analysis of the polymerization and thermal decomposition of an acrylic thermoplastic to establish a time–temperature–transformation (TTT) processability map. The process exhibits an induction period followed by rapid polymerization, indicating a complex mechanism. We introduce a novel methodology to quantify this induction period using differential scanning calorimetry (DSC) under both isothermal and dynamic conditions. The resulting kinetic parameters were used to construct the TTT map, which delineates a broad and practical processing window. This map enables precise control of the polymerization process, minimizes the risk of thermal degradation, and supports the efficient integration of acrylic resins into industrial composite manufacturing.

## Graphical abstract

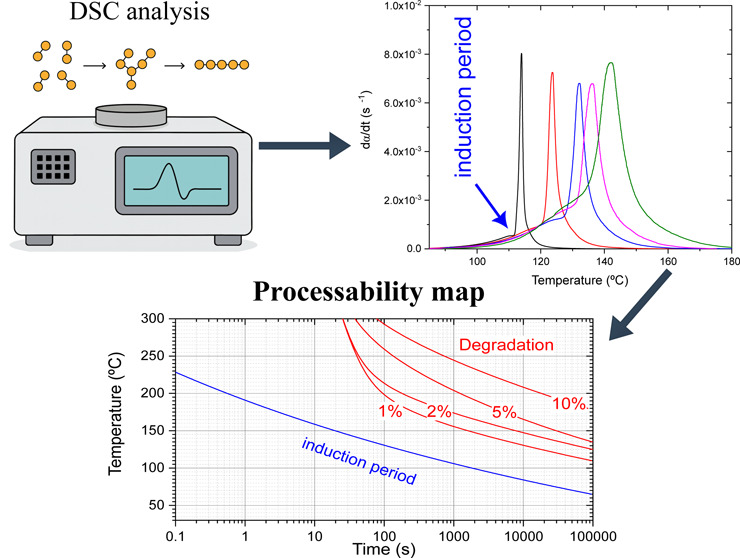

## 1 Introduction

Wind energy is essential to achieving Europe’s net-zero carbon target by 2050.^1^ However, the nonrecyclable composite blades of wind turbines are generating growing landfill waste, projected to reach 43 million tons globally by 2050—about 25% in Europe. This underscores the urgent need for recyclable blade materials.^2^

In this work, we investigate the polymerization and thermal degradation kinetics of Akelite, a fully recyclable thermoplastic resin patented by the Institute of Polymer Science and Technology (ICTP-CSIC).^3^ Akelite is a low-viscosity liquid acrylic resin, making it particularly suitable for the fabrication of fiber-reinforced polymer composites. Its low viscosity enables fiber impregnation at room temperature, allowing the use of conventional manufacturing techniques originally developed for thermosetting epoxy-based composites. Moreover, the resin exhibits excellent storage stability, remaining useable for over six months when kept below 25 °C. In addition to its favorable processing characteristics, Akelite demonstrates outstanding mechanical performance, positioning it as a promising eco-friendly alternative for structural applications such as wind turbine blades.^4^ To support its industrial implementation, this study focuses on characterizing both the polymerization and degradation kinetics of the resin.

To characterize the polymerization reaction and thermal degradation kinetics of Akelite, we employ isoconversional methods. These model-free approaches do not rely on predefined reaction mechanisms, making them more versatile and easier to apply than traditional model-based methods. Isoconversional techniques have been successfully used to study both polymerization and degradation processes in a wide range of polymeric systems.^5–14^ Furthermore, they provide accurate kinetic predictions, enabling the construction of time–temperature–transformation (TTT) diagrams, which are valuable tools for optimizing processing conditions.^15–19^

The main monomer in Akelite is methyl methacrylate (MMA), while benzoyl peroxide is used as an initiator to promote free-radical polymerization. This compound initiates a chain reaction through thermal decomposition, which involves the homolytic cleavage of the O–O bond, generating two free radicals. These radicals react with the monomers, initiating a propagation process in which the polymer chains grow by successive monomer additions. In the presence of a peroxide initiator, the bulk free-radical polymerization of MMA exhibits a characteristic sharp increase in reaction rate following an initial induction period, a phenomenon known as the Trommsdorff or “gel” effect.^20–22^

Initially, the low viscosity of the reaction medium facilitates rapid termination of macroradicals via recombination, which suppresses the polymerization rate. As the reaction progresses and the degree of polymerization reaches approximately 35–40%,^21,23^ the viscosity of the system increases significantly. This rise in viscosity restricts the mobility of macroradicals, thereby reducing termination events. Meanwhile, monomer molecules remain sufficiently mobile to sustain propagation. As a result, the reaction rate increases abruptly due to the accumulation of active radicals. The kinetics of the system is therefore mainly governed by the induction period, which corresponds to the time required for the suppression of macroradical termination.

Several authors have modeled the polymerization kinetics of MMA in the presence of initiators.^21,22,24–28^ However, accurately capturing the Trommsdorff effect remains a significant challenge due to the complexity of the underlying mechanisms and the need to determine numerous physical parameters. Moreover, all existing models incorporate free parameters that must be adjusted to accurately capture the reaction kinetics. Consequently, a considerable degree of empiricism underlies these models. Furthermore, the reaction kinetics is highly sensitive to the specific formulation of the resin, including factors such as initiator concentration, other additional monomers, and the presence of predissolved poly­(methyl methacrylate) (PMMA).

Model-free methods generally offer a simplified approach to reaction kinetics analysis. However, as will be demonstrated, the presence of the Trommsdorff effect significantly limits the effectiveness of isoconversional techniques in accurately characterizing the polymerization kinetics of MMA. Despite this limitation, we propose a straightforward methodology to quantify the induction period. Based on this characterization, we have developed a processing map that facilitates the identification of optimal manufacturing conditions while avoiding thermal degradation. These findings provide valuable guidance for wind turbine blade manufacturers, supporting the evaluation and adoption of recyclable materials as viable alternatives to conventional thermosetting systems.

## 2 Materials and Methods

### 2.1 Materials

The material under study is a combination of several acrylic monomers with methyl methacrylate (MMA) being the main monomer, and benzoyl peroxide is used as the initiator to promote bulk free-radical polymerization. The resin exhibits a maximum glass transition temperature of approximately 120 °C. During synthesis, the viscosity of the resin is adjusted to around 140 cP at room temperature, making it suitable for composite manufacturing processes via infusion. This resin has been patented by ICTP-CSIC under the name Akelite.^3^

To investigate the polymerization behavior, the resin is mixed with 3 wt % of initiator. To ensure homogeneity, the mixture is placed in an ultrasonic bath for 15 min. It is then transferred into hermetically sealed aluminum crucibles (40 μL capacity, TA Instruments) to prevent MMA evaporation during thermal analysis. The typical sample mass ranges from 0.5 to 1 mg, which minimizes the risk of self-heating and ensures proper thermal equilibration during differential scanning calorimetry (DSC) experiments.^29^

### 2.2 Differential Scanning Calorimetry

The polymerization reaction was monitored using a TA Instruments Q2000 differential scanning calorimeter (DSC). Experiments were conducted under both dynamic and isothermal conditions. Dynamic scans were conducted from 0 to 200 °C at heating rates ranging from 1.25 to 7.5 °C/min. Isothermal experiments were performed at temperatures between 90 and 120 °C, with durations varying from 1 h at 120 °C to 2.5 h at 90 °C. All measurements were conducted under an inert atmosphere, maintained by a continuous flow of high-purity nitrogen at 50 mL/min. An empty aluminum crucible, similar to that of the sample was used as a reference.

In isothermal experiments, the glass transition temperature (Tg) was used as an indicator of the degree of transformation. Following the isothermal step, each sample was subjected to a second DSC scan from 0 to 200 °C at a heating rate of 20 °C/min. The Tg was determined according to standard methodology, using the midpoint between the extrapolated onset and end point of the transition.

### 2.3 Thermogravimetric Analysis

Thermal degradation of the cured resin was analyzed using a Mettler Toledo thermobalance (model TGA/DSC1) via thermogravimetric analysis (TGA). A series of dynamic experiments were carried out from 50 to 700 °C at heating rates ranging from 1.25 to 20 °C/min, under an inert atmosphere maintained by a constant flow of high-purity nitrogen at 60 mL/min. Approximately 6 mg of sample was used in each test, placed in 150 μL Al_2_O_3_ crucibles.

### 2.4 Kinetic Analysis

The kinetic parameters of polymerization and degradation, including the apparent activation energy and the pre-exponential factor, were determined using Friedman’s isoconversional method.^30,31^ Isoconversional methods are based on the assumption that, for a given degree of conversion (α), the reaction rate depends solely on temperature.^32,33^ The Friedman method is expressed as

1 $${\left[\frac{d\ln\left(d\alpha /dt\right)}{{dT}^{-1}}\right]}_{\alpha }=-\frac{E_{\alpha }}{R}$$

where α is the degree of conversion (0 ≤ α ≤ 1), *T* is the absolute temperature, *R* is the universal gas constant, and *E*_α_ is the activation energy corresponding to a specific conversion degree.

For most thermally activated processes, the energy barrier remains nearly constant throughout the transformation.^34^ Therefore, variations in *E*_α_ should not be interpreted as changes in a single energy barrier, but rather as the result of multiple overlapping processes, each characterized by its own activation energy, or as a deviation from Arrhenius-type behavior.^5,6,35^ In such complex systems, *E*_α_ is best interpreted as an apparent activation energy rather than a true intrinsic value.

Friedman method is based on the integration of the eq 1

2 $$\frac{d\alpha }{dt}=Af\left(\alpha \right)\exp\left[-\frac{E_{\alpha }}{RT}\right]$$

Thus, the reaction kinetics can be described by two parameters that depend on the degree of transformation: the apparent activation energy *E*_α_ and the product Af­(α). The first step in determining these parameters is to discretize the degree of conversion using a small, constant increment Δα, such that α_*j*_ = *j*Δα. The objective of the kinetic analysis is then to determine the set of values *E*_*j*_ and Af­(α)|_*j*_. This can be achieved by performing experiments at different heating rates β_*i*_≡*dT*/*dt* (nonisothermal conditions) or at different fixed temperatures (isothermal conditions).^36,37^ For each heating rate β_*i*_, the transformation rate *d*α/*dt*|_*j*,*i*_ and the corresponding temperature *T*_*j*,*i*_ at a given degree of conversion α_*j*_, are determined. According to eq 2, a plot of $\ln{\left(\frac{d\alpha }{dt}\right)}_{j,i}$ versus 1/*T*_*j*,*i*_ should yield a straight line.

3 $$\ln{\left(\frac{d\alpha }{dt}\right)}_{j,i}=-\frac{E_{j}}{RT_{j,i}}+{\ln\left(Af\left(\alpha \right)\right)|}_{j}$$

From the slope and intercept of the linear fit, the values of *E*_*j*_ and Af­(α)|_*j*_, can be determined, respectively. Once these parameters are known, the evolution of the curing process under any given temperature program can be predicted by integrating eq 2

4 $$\alpha \left(t\right)=\int_{0}^{t}Af\left(u\right)\exp\left[-\frac{E\left(u\right)}{RT\left(u\right)}\right]du$$

where *u* is the degree of transformation.

## 3 Results and Discussion

This section presents the kinetic characterization of Akelite polymerization and thermal degradation. First, the polymerization kinetics is analyzed under both dynamic and isothermal conditions, with particular emphasis on characterizing the induction period associated with the Trommsdorff effect. Subsequently, the thermal degradation kinetics is examined. Finally, a time–temperature-transformation (TTT) processability map is constructed by integrating both processes.

### 3.1 Polymerization Kinetics

To determine the evolution of the degree of polymerization as a function of temperature, it is assumed that the heat flow released by the sample is directly proportional to the transformation rate. Therefore, the transformation rate *d*α/*dt* can be obtained directly from the DSC signal, normalized by the total enthalpy of the reaction^38^

5 $$\frac{d\alpha }{dt}=\frac{1}{\Delta H}\frac{dH}{dt}$$

where Δ*H* is the enthalpy obtained from the integration of the DSC curve and *dH*/*dt* is the heat flow measured by the DSC. Figure 1a shows the evolution of the rate of transformation and degree of polymerization obtained from DSC measurements made when the sample is heated at a constant rate, specifically five different heating rates have been analyzed: 1.25, 2.5, 3.75, 5, and 7.5 °C/min.

**Figure 1:**
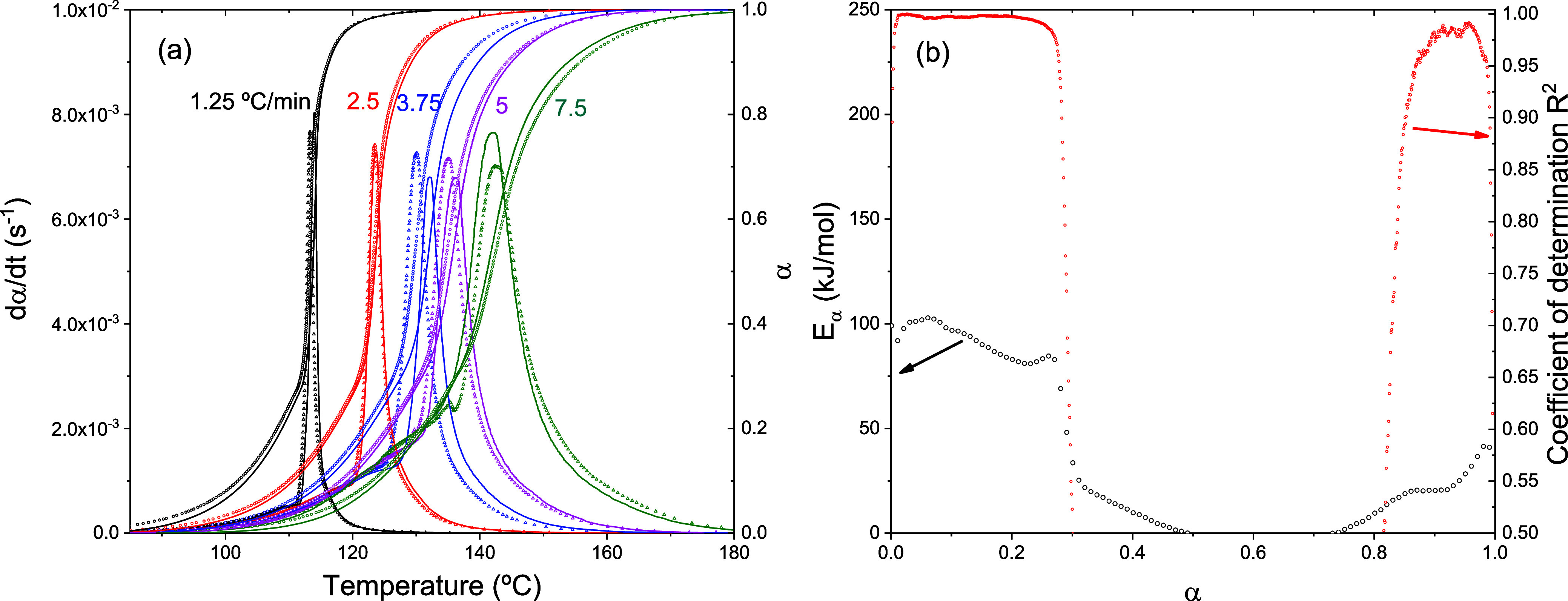
(a) Solid lines represent the rate of transformation (*d*α/*dt*) and the degree of polymerization (α) as functions of temperature, obtained by integrating DSC curves. Symbols indicate isoconversional predictions based on eq 4. (b) Activation energy of the polymerization reaction determined using Friedman isoconversional analysis (left axis), along with the coefficient of determination (*R* ^2^) of the linear fit (right axis).

The result of Friedman’s isoconversional analysis, based on eq 3, is presented in Figure 1b. From a degree of polymerization of approximately 30% (α = 0.3), the calculated activation energy drops to near zero, and the coefficient of determination shows that the data no longer follows a linear trend. This indicates that beyond this conversion level, the reaction is no longer governed by thermally activated kinetics and cannot be described by the isoconversional approach.

As a self-consistency check of the isoconversional model, eq 4 was used to simulate the polymerization behavior under the same dynamic conditions as those used in the experiments. The results, shown as symbols in Figure 1a, demonstrate good agreement with the experimental data, despite the limitations of the model beyond α = 0.3. This indicates that the isoconversional approach can still provide reliable predictions for nonisothermal polymerization processes within the explored heating rates range (1.25 to 7.5 °C/min).

The evolution of the polymerization rate, shown in Figure 1a, reveals two clearly distinguishable stages. The initial stage is characterized by a smooth and gradual increase in the process rate, followed by a sudden and pronounced acceleration. This behavior is a direct consequence of the Trommsdorff effect.

At the beginning of the reaction, the low viscosity of the resin facilitates efficient termination of macroradicals, maintaining a relatively low polymerization rate. However, after an induction period, the viscosity of the system increases significantly, which impedes the mobility of macroradicals and suppresses termination reactions. As a result, the concentration of active radicals rises sharply, leading to an abrupt increase in the polymerization rate. Beyond this point, the reaction is no longer governed by thermal activation, but rather by the accumulation of reactive species due to the inhibited termination.

A noteworthy observation is that the induction period corresponds to a specific degree of conversion.^21,23^ As shown in Figure 1a, the reaction kinetics exhibit a sudden acceleration at α = 0.3 across all experiments. This transition point marks the onset of the autoaccelerated regime.

Figure 1b further highlights this behavior by revealing three distinct kinetic regions. Below α = 0.3, the activation energy remains approximately constant at around 90 kJ/mol, and the high correlation coefficient indicates a strong linear relationship, consistent with thermally activated kinetics. Beyond this point, the activation energy drops sharply, and the correlation coefficient decreases significantly, suggesting a breakdown of the linear relationship between $\ln{\left(\frac{d\alpha }{dt}\right)}_{j,i}$ and 1/*T*_*j*,*i*_. This deviation confirms that the reaction is no longer governed by thermal activation, a feature that can be attributed to the autoaccelerated reaction.

To further investigate the mechanism change, we performed a master plot analysis using the normalized conversion function *f*(α)*g*(α)/*f*(0.5)*g*(0.5), which was obtained from the experimental data through the relationship *d*α/*dt*|_α_/*d*α/*dt*|_0.5_.^39^ This master curve is characteristic of the transformation mechanism. The results is shown in Figure 2, where a clear change in its trend is observed at α ≈ 0.3. This deviation indicates a transition in the polymerization mechanism, which is in agreement with the Friedman analysis shown in Figure 1. The consistency between both independent kinetic analyses reinforces the conclusion that the polymerization process undergoes a mechanistic change at this conversion level, marking the onset of the autoaccelerated stage.

**Figure 2:**
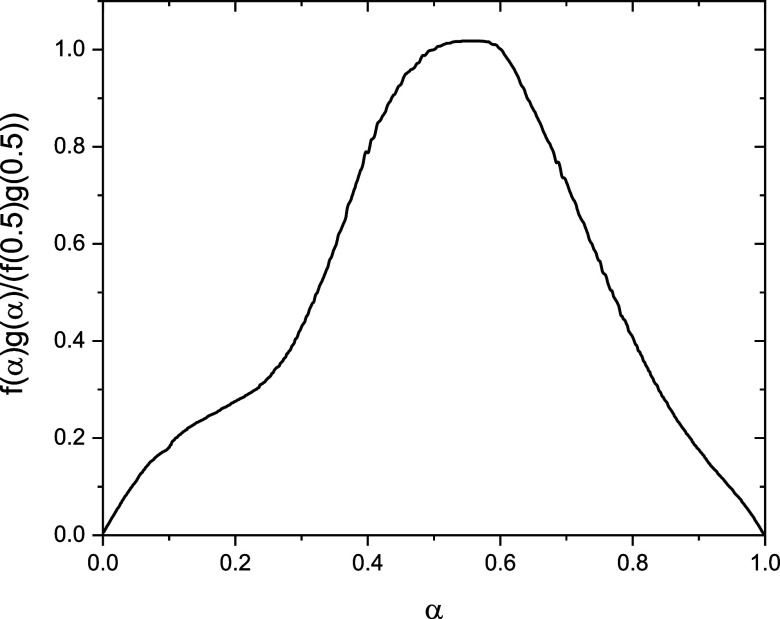
Master plot derived from DSC data at different heating rates (Figure 1a).

At higher degrees of conversion, particularly above α = 0.9, the correlation coefficient increases once again, approaching unity. This trend suggests a renewed thermal activation of the process, with the activation energy rising from 20 to 40 kJ/mol. Such behavior aligns with previous studies,^23,24^ which report that at advanced stages of conversion, the reaction rate declines because the reaction becomes diffusion-controlled.^23,24,40^

Therefore, to characterize the kinetics, it is essential to determine how the induction period depends on temperature. Given that this induction period corresponds to a fixed degree of transformation during the initial stage, it can be inferred that the induction period itself is thermally activated.

6 $$t_{i}=A_{i}\exp\left[\frac{E_{i}}{RT}\right]$$

To characterize the induction period, a series of isothermal experiments were conducted, as shown in Figure 3. According to eq 6, plotting the logarithm of the reaction onset time against the reciprocal of the absolute temperature yields a straight line. From the linear fit, both the activation energy and the pre-exponential factor can be determined.

**Figure 3:**
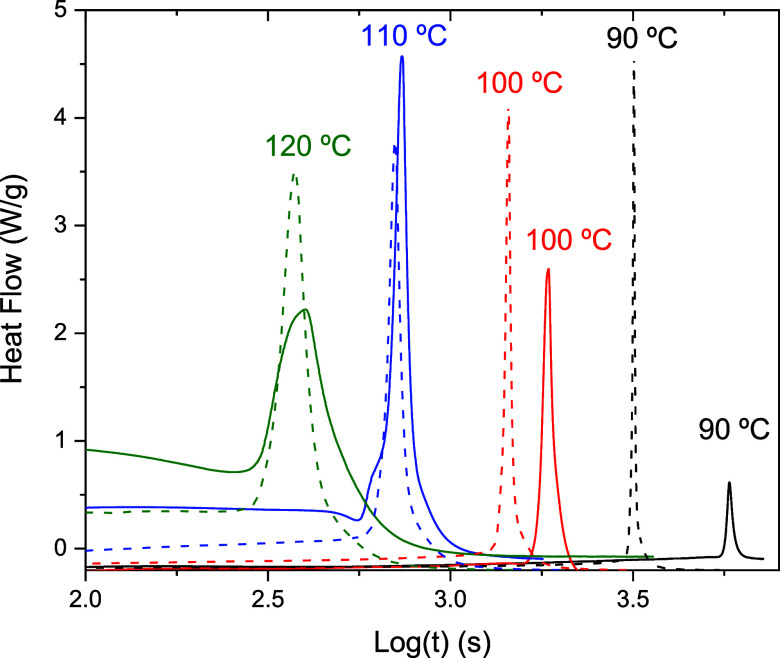
Continuous lines represent DSC isothermal measurements, while dashed lines correspond to isothermal isoconversional predictions derived from eq 4.

The resulting fit, presented in Figure 4, provides an activation energy of *E*_*i*_ = 118 kJ/mol, a pre-exponential factor of *A*_*i*_ = 4.22 × 10^–14^ s, and a coefficient of determination *R* ^2^ = 0.995. Based on the linear fit and eq 6, it is possible to predict the time required to initiate the chain reaction at any temperature. For instance, at 30 °C, the estimated induction time is approximately five months, which aligns with the known stability of Akelite at room temperature.

**Figure 4:**
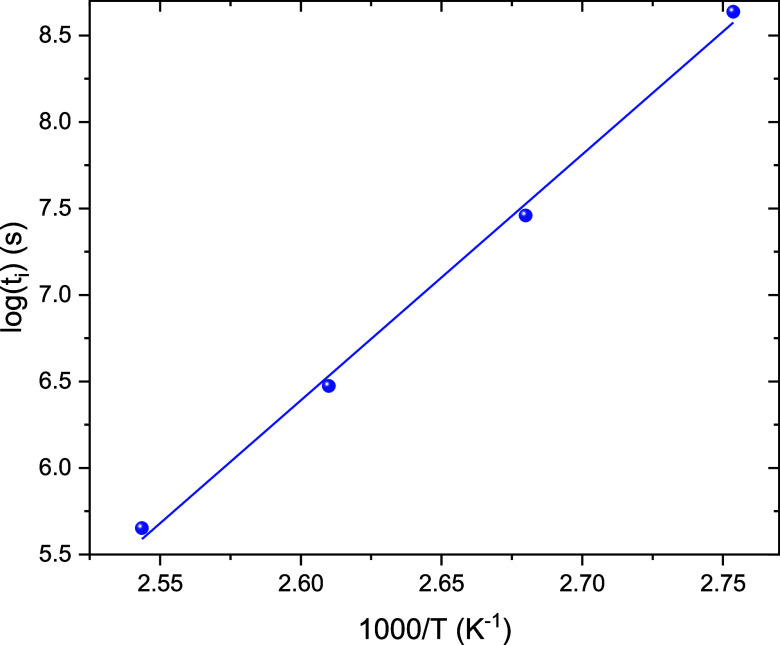
Plot of the logarithm of the reaction onset time versus the reciprocal of temperature.

Equation 6 can also be used to estimate the temperature at which the chain reaction initiates under nonisothermal conditions. Specifically, the contribution to the induction period during an infinitesimal time interval *dt* is given by

7 $$\frac{dt_{i}}{t_{i}}=\frac{dt}{A_{i}\exp\left[\frac{E_{i}}{RT}\right]}$$

Considering that the heating rate is β≡*dT*/*dt*, the temperature *T*_*i*_ at which the autoaccelerated stage starts is the solution of the equation

$$\frac{1}{A_{i}\beta }\int_{0}^{T_{i}}\exp\left[-\frac{E_{i}}{RT}\right]dT=1$$ 8

which can be reduced to

$$p\left(\frac{E_{i}}{RT_{i}}\right)=\frac{A_{i}\beta R}{E_{i}}$$ 9

where, *p*(*x*) is the integral of temperature^41^

$$p\left(x\right)=\int_{x}^{\infty }\frac{\exp\left(-u\right)}{u^{2}}du$$ 10

and $u=\frac{E_{i}}{RT}$ is the integration variable.

This integral does not have an exact solution, but there is a very precise sixth degree Padé series that allows its analytical evaluation.^31^

Using eqs 9 and ([Disp-formula eq10]), we calculated the temperature *T*_*i*_ corresponding to various heating rates and compared it with the temperature at which an abrupt change is observed in the DSC experiments shown in Figure 1a. The results are presented in Figure 5, which demonstrates that the kinetic parameters derived from the isothermal experiments successfully reproduce the behavior observed under constant heating rate conditions.

**Figure 5:**
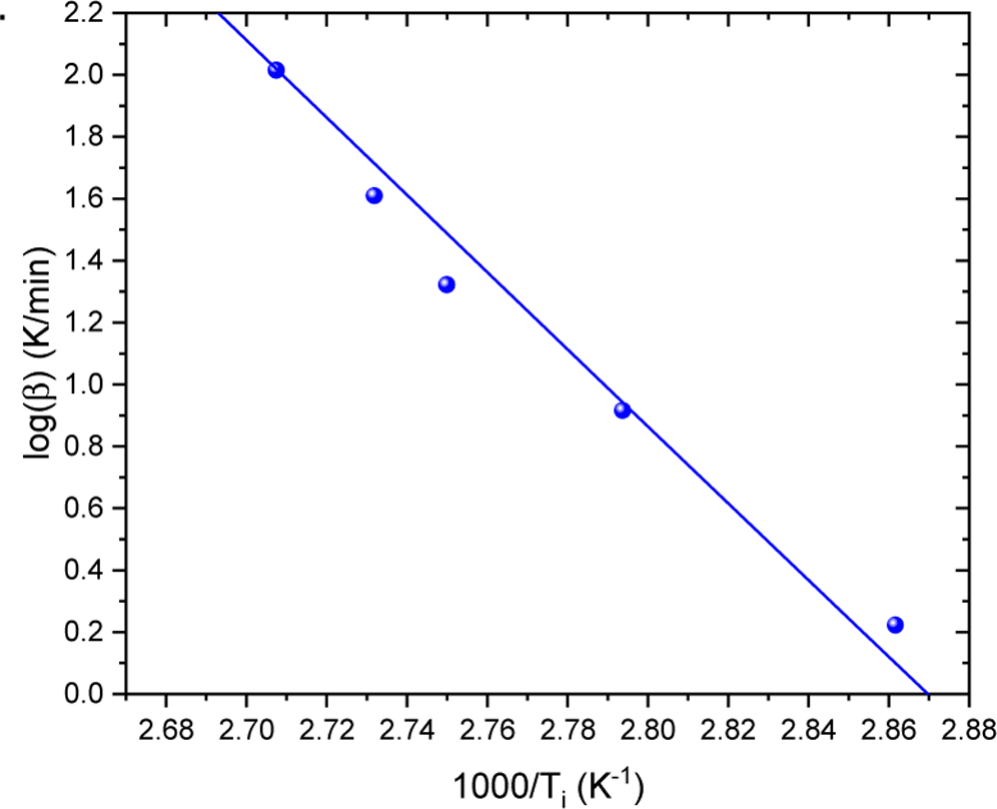
Logarithm of the heating rate as a function of the temperature at which the autoaccelerated reaction is triggered, as predicted by eqs 9 and ([Disp-formula eq10]) (solid line) and as observed experimentally (symbols).

It is important to emphasize that the line shown in Figure 5 is not a fitted curve, but rather a prediction based on eqs 9 and ([Disp-formula eq10]), using the values of *E*_*i*_ and *A*_*i*_ obtained from the isothermal data. The agreement between predicted and observed behavior confirms that the kinetic analysis of the induction period is reliable within the explored temperature range.

It is worth noting that eq 9 bears a formal resemblance to the relationship underlying the Flynn-Wall-Ozawa (FWO) method,^42,43^ which is widely used in thermal analysis for determining activation energies. The FWO method is based on the general rate equation for thermally activated processes under constant heating rate conditions.

11 $$\beta \frac{d\alpha }{dt}=Ae^{-E/RT}f\left(\alpha \right)$$

Integration of this eq 11 leads to

12 $$\beta g\left(\alpha \right)=A\int_{0}^{T}e^{-E/Rz}dz$$

where $g\left(\alpha \right)=\int_{0}^{\alpha }\frac{dx}{f\left(x\right)}$ .

When *g*(α) = 1, this expression becomes formally identical to eq 9. However, the physical meanings are fundamentally different: the FWO method describes the kinetics of thermally activated reactions through the conversion degree α, whereas eq 9 relates to the induction period preceding the reaction. Despite this conceptual difference, the mathematical similarity proves useful for understanding the linear behavior observed in Figure 5.

The FWO method achieves linearization through Doyle’s approximation of the temperature integral^44^

13 p(x)≈exp(−1.0518x−5.330)

Substituting this approximation and taking the natural logarithm yields

14 $$\ln\beta =-1.0518\frac{E}{RT}-5.33+\ln\left(\frac{AE}{Rg\left(\alpha \right)}\right)$$

This relationship predicts that a plot of ln β versus 1/*T* should exhibit a linear trend with slope −1.0518 *E*/*R*. By analogy, the same approximation justifies the linear behavior observed in Figure 5.

### 3.2 Kinetics of Thermal Degradation

TGA is particularly suitable for characterizing degradation kinetics because it monitors the mass evolution. In this case, it is usually assumed that the degree of degradation is linearly dependent on mass.

15 $$\varepsilon \left(t\right)=\frac{m_{i}-m\left(t\right)}{m_{i}-m_{f}}$$

where *m*_*i*_ and *m*_*f*_ are the initial and final sample masses, respectively.

Figure 6 a presents the degradation profiles at various heating rates, alongside the results of Friedman’s isoconversional analysis (Figure 6b). In this case, a good agreement is observed over a broad conversion range (ε > 0,06). Specifically, the comparison between the experimental curves and the predictions obtained from eq 4, shown in Figure 6a, confirms that the isoconversional model accurately reproduces the measured behavior. This demonstrates that the isoconversional approach provides a reliable framework for predicting the evolution of degradation under varying thermal conditions.

**Figure 6:**
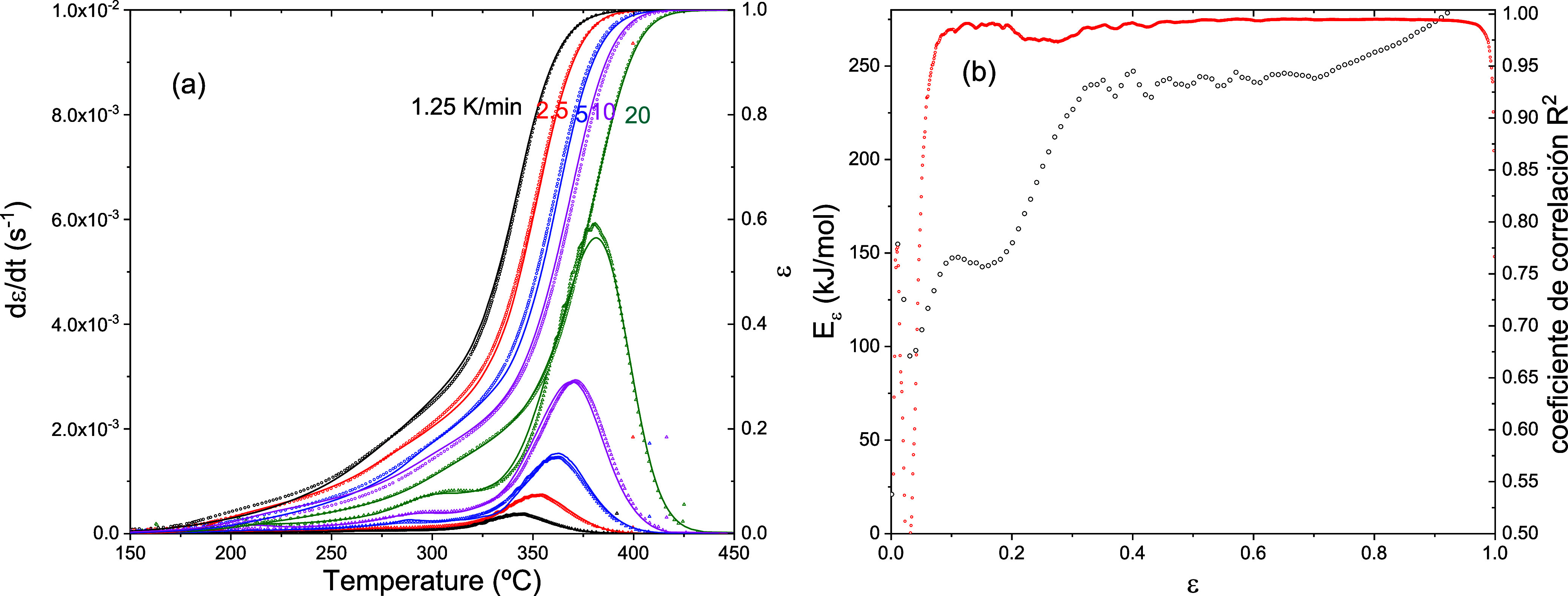
(a) Solid lines represent the degradation rate (*d*ε/*dt*) and the degree of degradation (ε) as functions of temperature, obtained from TGA measurements. Symbols indicate the isoconversional predictions derived from eq 4. (b) Activation energy of the degradation reaction determined via Friedman isoconversional analysis (left axis), along with the coefficient of determination (*R* ^2^) of the linear fit (right axis).

### 3.3 Processing Map

From the kinetic analysis we can construct a transformation time–temperature diagram (TTT) that takes into account the kinetics of polymerization and degradation. For this purpose and with the help of eq 6, we have determined the time required for the autoaccelerated reaction to occur. We have also included the time required for reaching a certain degree of degradation. The result is presented in Figure 7. The separation between polymerization and degradation, especially at elevated temperatures, is remarkable. This wide processing window significantly facilitates the use of Akelite resin for the manufacture of laminates. In particular, polymerization at elevated temperatures is in principle feasible for relatively short times.

**Figure 7:**
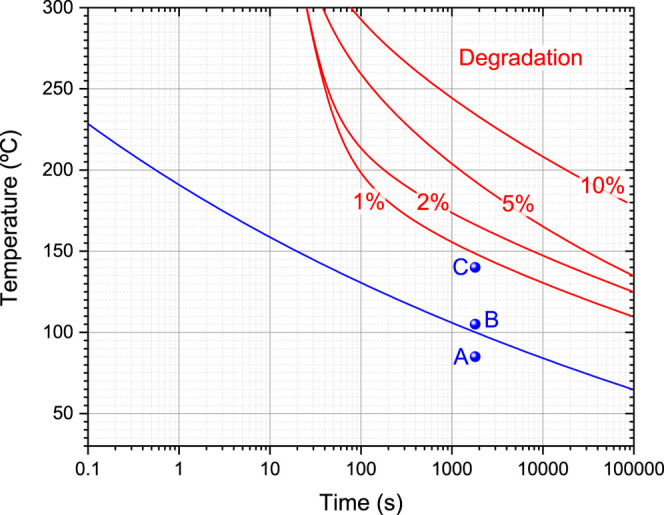
Isothermal processing map of Akelite resin. Red lines indicate the time required to reach a certain degree of thermal degradation at various temperatures. The blue line represents the incubation time at each temperature. Dots correspond to the isothermal polymerization cycles used to prepare samples A, B, and C.

To evaluate the reliability of the processing map, three samples were subjected to isothermal treatment for a fixed duration of 30 min at different temperatures: 85 °C (sample A), 105 °C (sample B), and 140 °C (sample C). We selected this time interval because, according to the TTT diagram prediction, sample A will not reach the incubation time, whereas samples B and C will. In addition, for sample C, even though the temperature is considerably higher, the prediction indicates that no significant degradation would occur. Samples B and C were selected to demonstrate the wide processing window available for Akelite. These isothermal curing conditions are represented as blue dots in Figure 7.

Figure 8 shows the DSC signals recorded during these polymersization cycles. Based on eq 6, the estimated incubation times for samples A, B, and C are 9050, 1100, and 45 s, respectively. For a 30 min curing cycle, sample A does not reach the autoaccelerated regime, as evidenced by the nearly flat DSC signal, indicating an almost negligible transformation rate throughout the cycle. In contrast, samples B and C exhibit sharp exothermic peaks, confirming that the curing process is completed in both cases. Notably, after the curing is complete, the DSC signal remains flat, suggesting that no degradation occurs under these conditions.

**Figure 8:**
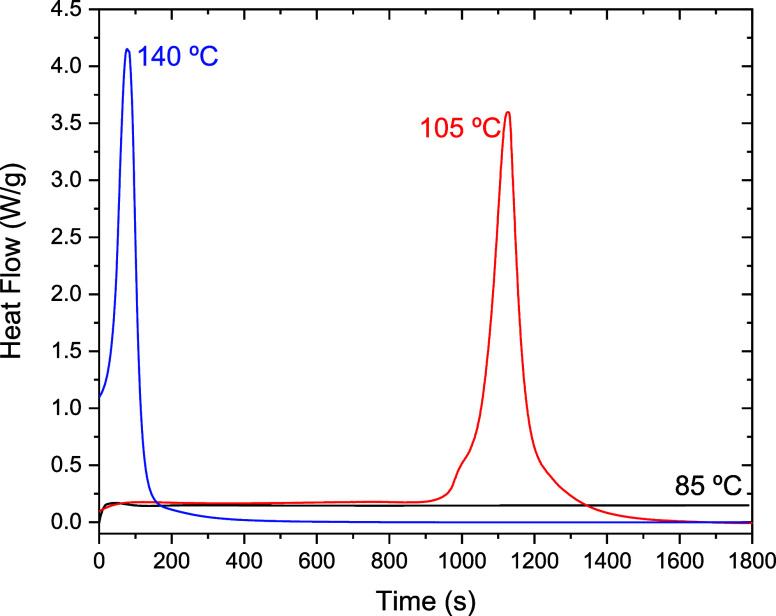
Isothermal DSC measurements corresponding to the curing cycles labeled (A) (85 °C), (B) (105 °C), and (C) (140 °C) as shown in Figure 7.

Visual inspection revealed that sample A had undergone almost no polymerization, whereas samples B and C were clearly solid, indicating a significant degree of polymerization. To confirm these observations and to quantify the extent of polymerization, a second measurement was performed using DSC. This involved heating the samples from room temperature to 200 °C at a rate of 20 K/min to determine their glass transition temperatures (Tg). As expected, no glass transition was detected for sample A. This is consistent with the reported Tg of MMA at −126 °C,^23,45^ which lies below the experimental temperature range, further indicating that no polymerization had occurred. In the same experiment, however, an exothermic signal was observed between 100 and 200 °C, corresponding to the polymerization process, as expected for a nonpolymerized sample. In contrast, samples B and C exhibited glass transition temperatures of 124 and 118 °C, respectively. Given that the maximum Tg for fully cured Akelite is approximately 120 °C, these results confirm that both samples B and C are effectively fully cured.

## 4 Conclusions

In this work, the polymerization and thermal degradation kinetics of Akelite resin were thoroughly analyzed, with particular attention to the role of the initiator in introducing an induction period that governs the overall reaction kinetics. Isothermal experiments enabled the characterization of the temperature dependence of this induction period, providing key insights into the incubation behavior associated with the Trommsdorff effect. The proposed methodology also allows predicting the temperature above which the polymerization reaction becomes self-accelerating under constant heating conditions. The proposed methodology for analyzing induction time offers a valuable framework for understanding the curing kinetics of resins influenced by this phenomenon.

In addition, thermal degradation kinetics were investigated through isoconversional analysis, yielding a detailed understanding of the resin’s thermal stability.

Based on these kinetic data, a Time–Temperature–Transformation (TTT) diagram was constructed to visualize the relationship between induction onset and degradation thresholds. This diagram serves as a practical guide for selecting suitable curing cycles, and the resulting processing map reveals a broad window within which composite panels can be manufactured safely and efficiently, minimizing the risk of thermal degradation and enhancing process reliability.
